# Tracking a Global Threat: a New Genotyping Method for Candida auris

**DOI:** 10.1128/mBio.00259-20

**Published:** 2020-03-10

**Authors:** Christina A. Cuomo, Alexandre Alanio

**Affiliations:** a Broad Institute of MIT and Harvard, Cambridge, Massachusetts, USA; b Institut Pasteur, Molecular Mycology Unit, CNRS UMR2000, National Reference Center for Invasive Mycoses and Antifungals (NRCMA), Paris, France; c Laboratoire de Parasitologie-Mycologie, Hôpital Saint-Louis, Assistance Publique-Hôpitaux de Paris (AP-HP), Paris, France; d Groupe Hospitalier Saint-Louis Lariboisière Fernand-Widal, Assistance Publique-Hôpitaux de Paris (AP-HP), Paris, France; e Université de Paris, Paris, France

**Keywords:** *Candida auris*, genome analysis, genotyping, short tandem repeats

## Abstract

Over the past decade, Candida auris has emerged as an urgent threat to public health. Initially reported from cases of ear infections in Japan and Korea, C. auris has since been detected around the world. While whole-genome sequencing has been extensively used to trace the genetic relationships of the global emergence and local outbreaks, a recent report in *mBio* describes a targeted genotyping method as a rapid and inexpensive method for classifying C. auris isolates (T. de Groot, Y. Puts, I. Berrio, A. Chowdhary, and J. F. Meis, mBio 11:e02971-19, https://doi.org/10.1128/mBio.02971-19, 2020).

## Abstract (precis)

Over the past decade, Candida auris has emerged as an urgent threat to public health. Initially reported from cases of ear infections in Japan and Korea, C. auris has since been detected around the world. While whole-genome sequencing has been extensively used to trace the genetic relationships of the global emergence and local outbreaks, a recent report in *mBio* describes a targeted genotyping method as a rapid and inexpensive method for classifying C. auris isolates (T.

## COMMENTARY

The fungal species Candida auris is an emergent public health threat, classified among the most urgent antibiotic-resistant threats by a 2019 Centers for Disease Control and Prevention report (1). Initially described only a decade ago as a species associated with ear infections (2), C. auris has since been detected as a skin commensal yeast as well as the causative agent of systemic infections and has been reported in an expanding list of countries.

While as of yet affecting a smaller number of patients than other *Candida* species, such as C. albicans and C. glabrata, C. auris has posed major new challenges for the treatment and control of these infections. Unlike other *Candida* species, C. auris has caused clusters of cases or outbreaks which have been difficult to control, as this species persists in hospital environments, where it is difficult to eradicate. Infections also have more limited options for treatment due to the high rate of antifungal drug resistance; this species is nearly always resistant to azole drugs and is frequently resistant to additional classes of antifungal drugs (3). Of grave concern, isolates resistant to all three major antifungal drug classes used in clinical practice have also been detected. While invasive infections by other *Candida* species currently have a much larger impact and drug resistance in these organisms poses a serious threat (1), the intrinsic properties of C. auris have raised concerns about the potential for the expanded impact of difficult-to-treat cases.

Accurate identification of C. auris infections required updating of diagnostic approaches and development of new molecular methods (3). Distantly related to the most commonly observed *Candida* species infecting humans, C. auris was initially frequently misclassified as a related species, Candida haemulonii. Updating matrix-assisted laser desorption–ionization time of flight (MALDI-TOF) mass spectrometry and sequence databases to include C. auris and the use of sets of known and characterized isolates for testing have enabled improvement of methods for clinical species identification. However, in routine laboratories, such approaches cannot more finely map isolate relationships nor specifically track important mechanisms of drug resistance.

Whole-genome sequencing was critical in discerning the unusual population structure of now five distinct phylogenetic clades based on the analysis of isolates from around the world. While initial typing of this new species was based on sequencing of ribosomal genes (2), genome sequencing established that there are five well-separated genetic clusters or clades; these clades have been named for the initially reported geographic regions (South Asia, East Asia, Africa, South America, and Iran) and also assigned numbers (clades I, II, III, IV, and V, respectively) (4, 5). Most global cases, including those linked to outbreaks, have been assigned primarily to three predominant clades (I, III, and IV) (6–8). In investigating outbreaks, genome sequencing of isolates from patient clusters has provided evidence for the close genetic relationship of epidemiologically linked cases. However, genetic diversity both within a patient and across the isolates within an outbreak has also been observed, suggesting a simmering threat of infections. The large number of sequenced isolates from around the world has also provided evidence of infections acquired during travel, often associated with hospital stays in other countries. With genomes of over 700 isolates publicly available in GenBank, a deep resource of data is available for genomic comparisons.

These genomes have been further mined by correlating genomic changes in known mutation hot spots, including drug target loci, with resistance data to identify resistance mechanisms. Amino acid substitutions associated with azole and echinocandin resistance have been identified in most resistant isolates (4, 7, 8). Different point mutations, such as those observed in the azole target *ERG11*, are observed in each clade. Amphotericin resistance is not yet as well characterized because this drug does not rely on inhibition of a specific enzyme, and how well estimated *in vitro* breakpoints correspond to true clinical resistance is more debated. Validation of the growing genomic catalog of resistance sites will help map the basis of resistance to different drugs. While resistance to the front-line treatment of invasive candidiasis, echinocandins, is rarely observed (1 to 1.25% of cases), this will be important to closely monitor, as will the susceptibility of isolates to all antifungal drugs, as any increase in echinocandin resistance and in the emergence of multidrug-resistant isolates will raise the urgency of evaluating recently developed drugs with new mechanisms of action. Drug resistance will be important to diagnose rapidly after species identification and to monitor during treatment, both to select and adapt treatments that will be effective and to track how multidrug resistance is potentially arising.

While whole-genome sequencing has been widely used to examine the relationship of isolates from various pathogenic fungi, including C. auris, targeted genotyping methods widely used for other pathogenic fungi may also be applied. Genotyping methods are implemented for various purposes, such as outbreak investigation, phylogenetic studies, or evaluation of the intrinsic diversity within a given species. The two widely used PCR-based methods include multilocus sequence typing (MLST) and microsatellite length polymorphism (MLP) typing using short tandem repeats (STRs) (9). Multilocus sequence typing typically targets genic regions, which are amplified, sequenced (mainly by Sanger sequencing), and classified by unique allele sequence types based on mutations/polymorphisms acquired during evolution. STR typing instead focuses on repetitive sequences that are subject to other mechanisms of variation, such as amplification or contraction of repeated di-, tri-, and quadri (or more)-nucleotides. STRs do not follow the same molecular clock underlying spontaneous mutations, such as single nucleotide polymorphisms (SNPs), and therefore have the potential to provide higher resolution of genetic relationships.

A new report in *mBio* by de Groot et al. describes the development of an STR typing approach for Candida auris (10). STR markers were initially identified from a search across reference genome assemblies generated for each of the initially described four major clades (11). From 23 candidate STRs, a set of 12 STRs (di-, tri-, and nona-nucleotide repeats) that were present in all clades and showed copy number variation across an initial set of 10 isolates from 4 clades were selected. While genomic location was not a criterion in the selection, four chromosomes are represented by the six genic STRs based on clade IV reference locations; although the locations of the six intergenic STRs are not described, these sites could be easily mapped from the sequence. The 12 loci can be amplified in four multiplex PCRs, followed by sequencing, amplicon length detection, and analysis. When this assay was run on a set of 444 isolates that included large sets from single hospitals and outbreaks, a total of 40 genotypes were detected. A minimal spanning tree connected these into the five known clades, supporting the idea that STRs may be used for clade assignment in C. auris. In comparison with WGS data, the STR genotypes detected largely correspond to those found by phylogenetic analysis of whole-genome SNP data. However, relationships within clades appear to have some discrepancies from those supported by genomic data, as STR genotypes are not all monophyletic.

The best applications of STR analysis for C. auris need elaboration, as do considerations of when to use this approach in place of genome sequencing. The higher resolution of whole-genome sequencing may be required when reconstructing isolate relationships, such as within an outbreak cluster. However, STR analysis may be helpful for initial classification of large sets of isolates at a subspecies level; this STR assay can assign isolates to one of the five known clades and give a preliminary idea of the genetic variability within a study set. As the clinical population structure of C. auris is quite different from that of other pathogens where STR typing is applied, adding markers to more finely resolve relationships within a clade and resolve discrepancies with genome data would be needed. Additional development may also prioritize clade discriminatory markers; several markers in the current panel have the same allele in clade I and III isolates, and none appear to be conserved in and unique to all isolates from a clade. Additional development might also examine STR variability across a more genetically diverse set of isolates, such as by screening the over 700 genomes in the NCBI database, selecting those that are not part of clonal clusters. This would provide needed information about the mutation rates of different STR markers, which might help guide their selection. The use of a set of reference isolates that is easily available to researchers around the world is also critical to include for standardizing STR clade assignments by different groups.

Comparison across studies will require development of a centralized database that includes quality standards. In addition to genotypes being shared, the raw data of the capillary electrophoresis could be curated, as is done for various MLST databases. Recommended best practices will also help yield more comparable studies. These should include standardization of DNA input requirements and inclusion of an allelic ladder, which have been shown to impact typing results (12). Simplifying analysis procedures is also needed; currently, expertise and specialized software are required to generate genotypes. The use of different software to construct minimum spanning trees may affect results and therefore the relationships between genotypes or isolates. Providing data that can be reanalyzed, as for the standard of depositing raw whole-genome sequences, may enable greater portability and extensibility. However, the comparability of data generated using different types of sequencers and software would need to be addressed.

While whole-genome sequencing is being widely applied at a global scale for C. auris, the importance of widely tracking this emerging species should motivate further evaluation of targeted methods, such as STR analysis. Further development might target markers to detect clinically relevant genotypes. The *mBio* report by de Groot et al. provides an initial approach for STR analysis that may be adapted as an easy screening tool for C. auris, supported by further development to account for wider genetic diversity, to allow for standardization across sites, and to support data sharing.
